# Reciprocal suppression between Zbtb1 expression and IL‐7Rα signalling during T‐cell development

**DOI:** 10.1111/jcmm.13663

**Published:** 2018-06-08

**Authors:** Xin Cao, Xiao‐xia Ma, Jiang‐long Du, Yan Zeng, Xian‐yu Zhang, Ying Lu, Yu‐jia Xue, Peng Ma, Qiu‐yan Chang, Lin‐jie Li, Xue‐yan Zhou, Kui‐zheng Cai, Damian Kovalovsky, Zhong‐ren Ma

**Affiliations:** ^1^ Key Laboratory of Bioengineering & Biotechnology of State Ethnic Affairs Commission Engineering & Technology Research Center for Animal Cell, Gansu College of Life Science and Engineering Northwest Minzu University Lanzhou China; ^2^ Experimental Immunology Branch National Cancer Institute National Institutes of Health Bethesda MD USA; ^3^ State Key Laboratory of Veterinary Etiological Biology Lanzhou Veterinary Research Institute Chinese Academy of Agricultural Sciences Lanzhou China; ^4^ Ministry of Education Key Laboratory of Molecular Microbiology and Technology Nankai University Tianjin China

Dear Editor,

The BTB‐ZF (broad‐complex, tramtrack and bric‐à‐brac–zinc finger) proteins play essential roles in the development of the immune system.^1^ Transcriptional repressor Zbtb1is one of the BTB‐ZF members essential for lymphocyte development^2^, ^3^ and NKp46^+^ ROR‐gamma‐T^+^ innate lymphoid cell (ILC3) development.^4^ Although the mechanisms by which Zbtb1 promote lymphoid development have been investigated,^5^, ^6^ many questions are still unsolved, especially for T‐cell development. T cells require IL‐7 signalling throughout their life, including maturation, differentiation and survival in peripheral lymphoid tissues.^7^ Both B and T cells developmental block were seen in the mice deficient for IL‐7 receptor α‐chain (IL‐7Rα).^8^ Interestingly, enforced expression of Bcl‐2 restored T‐cell development but not B cell in IL‐7Rα^−/−^ mice,^9^, ^10^ however, Bcl‐2 overexpression in the ScanT (Zbtb1 mutant) mice restored B cell but only early T‐cell development.^5^ In this study, the interplay between Zbtb1 and IL‐7Rα was dissected during T‐cell development.

At first we decided to evaluate the regulation of Zbtb1 expression by IL‐7Rα signalling during T‐cell development in vitro. We chose D1 cell line as a simplified research model, which was an IL‐7 (interleukin‐7)‐dependent thymic cell line derived from a p53^−/−^ mouse. According to the surface maker expression by flow cytometry, D1 is a DN1(double negative, stage 1)‐like cell line, which is CD44^+^CD25^−^IL‐7Rα^+^(Figure S1A). D1 cells were maintained in complete medium with 10 ng/mL IL‐7. To test the impact of IL‐7Rα signalling on Zbtb1 expression, D1 cells were starved overnight by IL‐7 deprivation, and then re‐stimulated with 10 ng/mL IL‐7. The Zbtb1 mRNA level reached the peak after IL‐7 deprivation overnight. However, the Zbtb1 transcripts down‐regulated after IL‐7 re‐stimulation, with the lowest level at 4 hours adding IL‐7 (Figure 1A). In order to investigate the effect of IL‐7Rα signalling on Zbtb1 expression in vivo, we utilized the mice deficient for IL‐7Rα and IL‐7Rα^−/−^ compound with IL‐7Rα transgene under the control of human CD2 promoter (T cell‐specific). We sorted double positive (DP), CD4 single positive (SP) and CD8SP thymocytes from wild‐type C57BL/6 and IL‐7Rα mutant mice mentioned above (Figure 1B), then performed RT‐qPCR assay. We found Zbtb1 expression level were elevated in all cell types in IL‐7Rα^−/−^ compared to wild‐type. However, Zbtb1 transcripts were down‐regulated in all cell types of mice with IL‐7Rα^−/−^ compound with IL‐7Rα transgene. These data suggest that the transcription of Zbtb1 can be negatively regulated by IL‐7Rα signalling.

**Figure 1:**
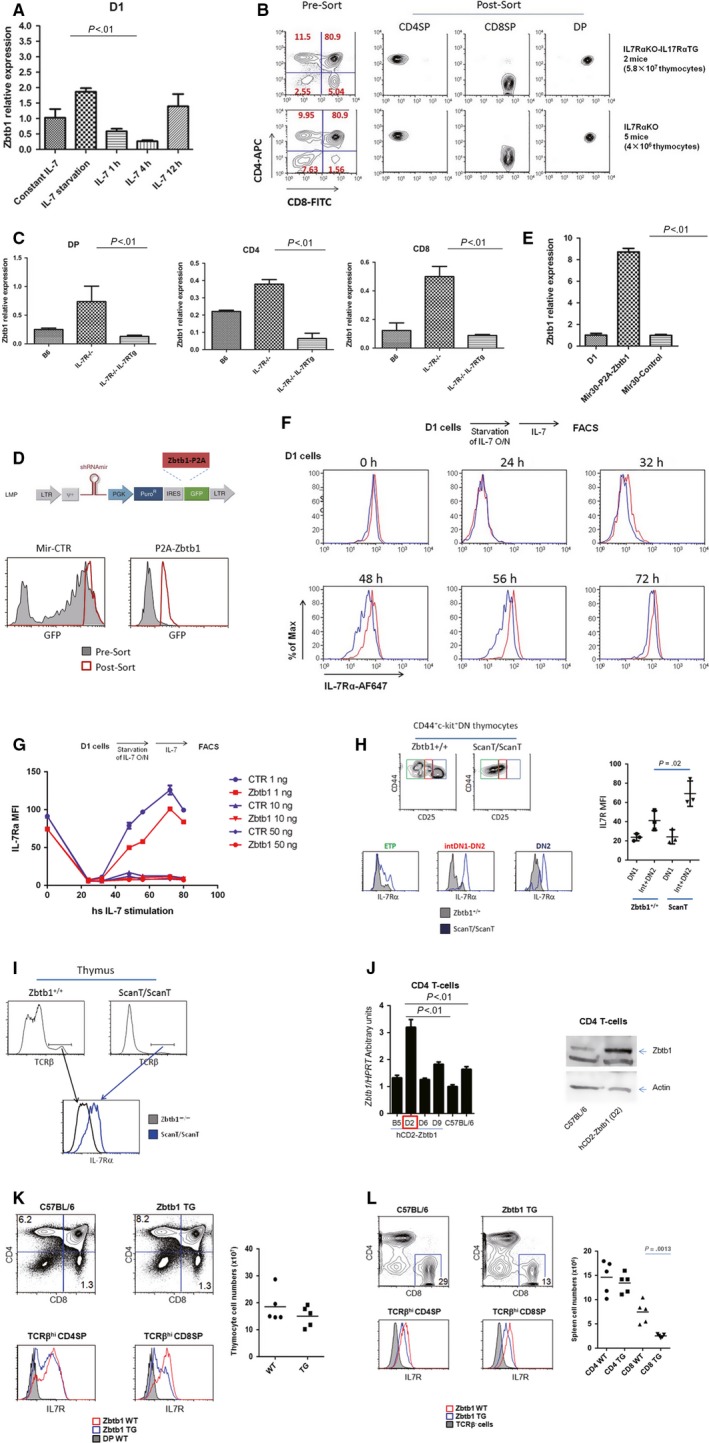
Zbtb1 and IL‐7Rα signalling mutually regulate each other both in vitro and in vivo. A, D1 cells were maintained in complete medium with 10 ng/mL IL‐7 or deprived of IL‐7 overnight and re‐stimulated with IL‐7 for 1 h, 4 h and 12 h. Zbtb1 transcripts were analysed by RT‐qPCR in various conditions. HPRT were served as internal control. B, DP, CD4SP and CD8SP thymocytes were sorted from mice with different genotypes as indicated. C, Zbtb1 transcripts from thymocytes mentioned in (B) were detected by RT‐qPCR. D, D1 cells were transduced by retroviral vector Mir‐CTR or Zbtb1 overexpressing retrovirus Mir‐P2A‐Zbtb1. Stable cell lines were generated by sorting GFP ^+^ cells. E, Zbtb1 overexpression in D1 stable cell line was confirmed by RT‐qPCR. F, G, Two stable D1 cell lines with or without Zbtb1 overexpression were deprived of IL‐7 overnight and re‐stimulated with various concentration of IL‐7 for different period of time. Surface IL‐7Rα expression in each condition was detected by flow cytometry. The representative data of D1 cells re‐stimulated with 1 ng/mL IL‐7 after starvation were shown in (F). H, I, The surface IL‐7Rα expression of different subpopulation of thymocytes in wild‐type and ScanT mice were analysed by flow cytometry. J, The overexpression of Zbtb1 in the D2 line of hCD2‐Zbtb1 transgenic mice was confirmed by RT‐qPCR and Western blot. K, The total numbers of thymocytes between wild‐type and Zbtb1 transgenic mice were comparable. The surface IL‐7Rα expression in CD4SP and CD8SP thymocytes of wild‐type and Zbtb1 transgenic mice were evaluated by flow cytometry. L, The total numbers of CD4 and CD8 T cells in spleen were compared between wild‐type and Zbtb1 transgenic mice. The surface IL‐7Rα expression in CD4SP and CD8SP TCRβ^hi^ spleenocytes of wild‐type and Zbtb1 transgenic mice were evaluated by flow cytometry

Next we investigated the effect of Zbtb1 on IL‐7Rα expression. We transduced the D1 cells with either retroviral vector Mir‐CTR or Zbtb1‐overexpressing retrovirus, and sorted the GFP^+^ cells to establish the stable cell line (Figure 1D). The Zbtb1 overexpression in Mir30‐P2A‐Zbtb1 stable cell line was confirmed by RT‐qPCR (Figure 1E). We deprived IL‐7 from culture medium of D1 cells overnight, re‐stimulated with different concentration of IL‐7, and then analysed surface IL‐7Rα level at different time post‐stimulation. We found that Zbtb1 overexpression can down‐regulate IL‐7Rα under sub‐optimal IL‐7 concentration (1 ng/mL) (Figure 1F,G). In order to address the impact of Zbtb1 on IL‐7Rα during T‐cell development in vivo, we gated different sub‐population in thymocytes (Figure S1B). Consistent with previous report, IL‐7Rα level in wild‐type thymus up‐regulated from ETP to DN2a stage, gradually reduced to undetectable level in DP stage, then recovered in SP stage (Figure S1C). Interestingly, we found that ScanT thymocytes expressed higher level of IL‐7Rα compared to wild‐type thymocytes during T‐cell development, including ETP, intermediate DN1 to DN2 stage (intDN1‐DN2) and DN2 stage (Figure 1H). The cell size of ETP, intDN1‐DN2 and DN2 thymocytes seemed to be smaller in ScanT mice, indicating that they are less proliferative than that of wild‐type (Figure S1D). The IL‐7Rα level of TCRβ^hi^ thymocytes in ScanT mice was also higher than that of their wild‐type littermates, although the percentage of TCRβ^hi^ thymocytes in ScanT mice was severely reduced (Figure 1I). Furthermore, we generated Zbtb1 transgenic mice in which expression of Zbtb1 was driven by T‐cell specific hCD2 promoter. Among four transgenic mouse line generated, only D2 line successfully overexpressed Zbtb1 in CD4 T cells compared to C57BL/6 in both mRNA and protein level (Figure 1J). The total number of thymocytes was comparable between wild‐type and hCD2‐Zbtb1 transgenic mice. However, the IL‐7Rα level in CD4SP and CD8SP thymocytes of transgenic mice was lower than that of wild‐type mice (Figure 1K). In the spleen, both CD4SP and CD8SP cells from transgenic mice expressed lower level of IL‐7Rα than that of wild‐type mice. Surprisingly, the number of CD8SP spleenocytes in hCD2‐Zbtb1 transgenic mice was greatly reduced, while the number of CD4SP spleenocytes was comparable (Figure 1L). The reduction in the number of CD8SP spleenocytes in ScanT mice was not due to unregulated expression of Bcl2, although CD8SP spleenocytes had lower level of IL‐7Rα in ScanT mice compared to wild‐type counterpart (Figure S1E).

Altogether, our results suggest that Zbtb1 and IL‐7Rα signalling can regulate each other during T‐cell development. IL‐7Rα signalling negatively regulate Zbtb1, and vice versa. Detailed molecular mechanisms by which Zbtb1 regulate IL‐7Rα expression in T cells are still under investigation.

## CONFLICT OF INTEREST

The authors confirm that there are no conflicts of interest.

## Supporting information


** **
