# Supplementary figures and images for: Reciprocal suppression between Zbtb1 expression and IL‐7Rα signalling during T‐cell development
Source: J Cell Mol Med. 2018 Jun 8;22(8):4012–5. doi: 10.1111/jcmm.13663 (PMC6050490; doi:10.1111/jcmm.13663)

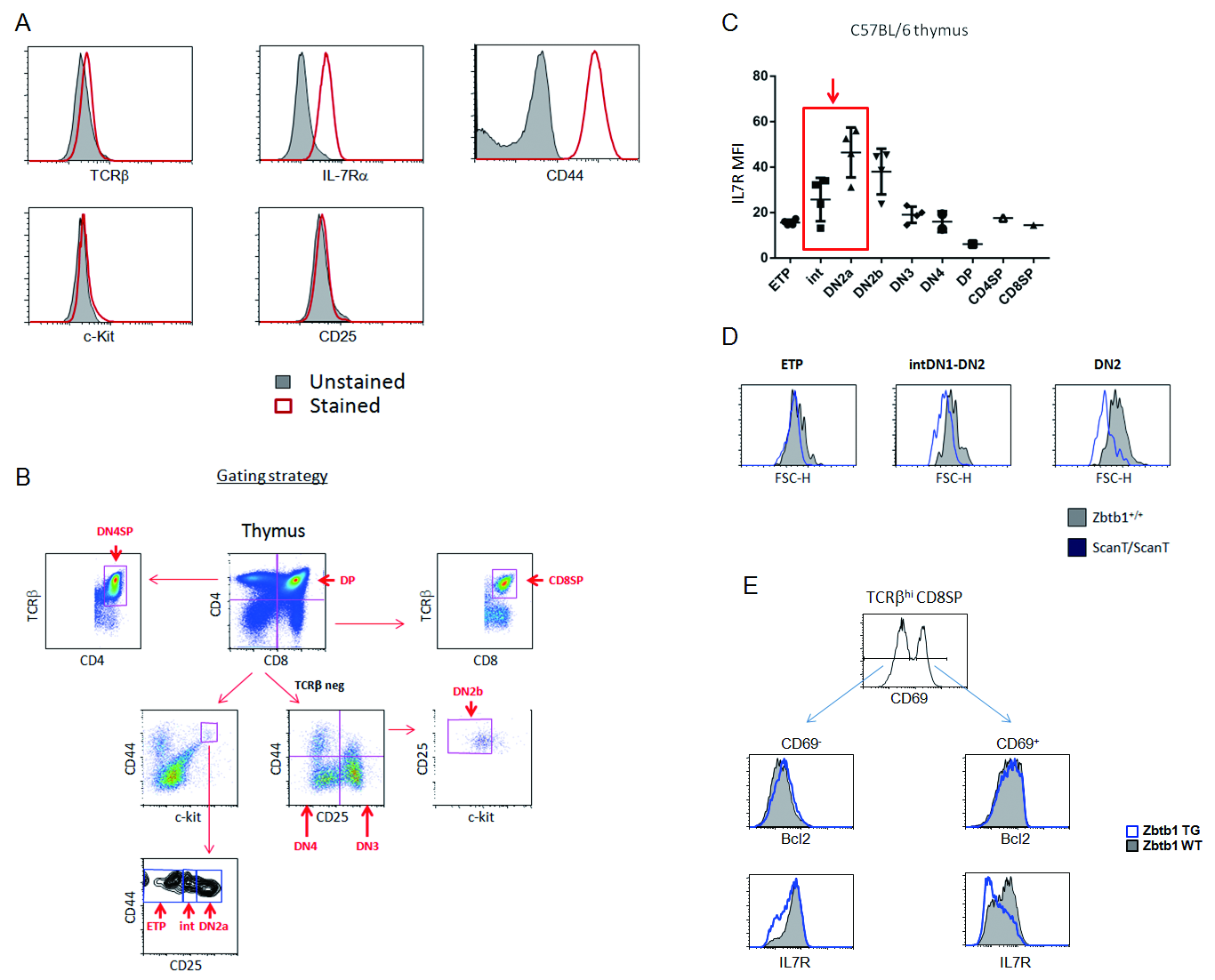

Supplement: Supplementary file 1 [file JCMM-22-4012-s001.tif]
